# Metabolic syndrome in premenopausal and postmenopausal women with type 2 diabetes: loss of protective effects of premenopausal status

**DOI:** 10.1186/s40200-014-0102-5

**Published:** 2014-11-23

**Authors:** Manouchehr Nakhjavani, Mehrnaz Imani, Mehrdad Larry, Arash Aghajani-Nargesi, Afsaneh Morteza, Alireza Esteghamati

**Affiliations:** Endocrinology and Metabolism Research Center (EMRC), Vali-Asr Hospital, Tehran University of Medical Sciences, P.O. Box: 13145–784, Tehran, Iran

**Keywords:** Metabolic syndrome, Menopause, Women, Cardiovascular disease

## Abstract

**Background:**

Diabetes is probably responsible for worsening of metabolic syndrome (MetS)components. The aim of the present study was to compare the components of MetS between premenopausal and postmenopausal women with type 2 diabetes (T2DM).

**Method:**

In this cross sectional study, we studied 639 women with T2DM that were divided in pre-menopausal (n = 221) and post-menopausal (n = 418) group. They were selected from participants of a diabetes clinic and assessed for MetS and its components. All MetS components were evaluated to follow age and duration of diabetes adjusted according to the ATP III criteria.

**Results:**

The mean ages of pre-menopausal and post-menopausal were 43.33 ± 0.47 and 60.35 ± 0.38 years, respectively. MetS was defined for 88.3% of total subjects (87.5% and 87.7% in pre-menopausal and post-menopausal women with T2DM respectively). Systolic blood pressure (SBP) and waist circumference (WC) were significantly higher in post-menopausal women with T2DM in comparison with pre-menopausal ones. There were no significant differences in triglyceride (T.G) level, diastolic blood pressure (DBP) and high density lipoprotein cholesterol (HDL-C) between the two groups. Myocardial infarction (MI) occurred in 1% total subjects (1.3% and 1.8%) in pre-menopausal and post-menopausal women with T2DM, respectively (p = 0.21).

**Conclusion:**

Worsening of MetS and its components except for SBP and waist circumference has been shown in pre-menopausal women with T2DM similar to post-menopausal ones. The observed differences may be explained by increasing age. With respect to increasing of myocardial infarction in premenopausal subjects, we suggest that diabetes can abolish the protective effects of premenopausal status for MetS and MI.

## Introduction

Metabolic syndrome (MetS) is a cluster of characteristics related to insulin resistance that increase the risk of cardiovascular disease (CVD) and type II diabetes mellitus (T2DM) [1]. Global prevalence of MetS is predicted to increase in the global population over coming years [2].One study reported a MetS prevalence of 58% in the general population of Iran [1]. It is possible that an increasing global prevalence of MetS will contribute to an increase in the number of cases of diabetes worldwide [3]. Furthermore, post-menopausal status presents with an increase in MetS characteristics and CVD, which are principally caused by the loss of female sex hormones [4]. Some studies have reported that menopausal status is an independent predictor of MetS among women [1,5]. However, no studies have assessed MetS and its components in a diabetic population of women. There is lack of data on the relationship between MetS and its components and menopausal status in diabetic women. The aim of the present study was to assess and compare MetS and its components in pre-menopausal and post-menopausal women with T2DM.

## Material and methods

We performed a clinical, cross-sectional study. The study population consisted of 639 participants, including 221 pre-menopausal women with T2DM and 418 post-menopausal women with T2DM. Patients were recruited from the diabetes clinic of Vali-Asr Hospital, which is affiliated with Tehran University of Medical Sciences.

We defined menopausal status according to the definition of the Reproductive Aging Workshop (2011) [6]. Pregnant women, patients with a mental or physical disability and women who had used hormone replacement therapy or oral contraceptive pills within the previous six months were excluded.

MetS was defined according to National Cholesterol Education Adult Treatment Panel III (NCEP ATP III) criteria [7]. As detailed in the NCEP ATP III report, participants having three or more of five following criteria were defined as having the metabolic syndrome: high blood pressure (>130/>85 mmHg) or known hypertensive patients, elevated fasting blood glucose (FBS >100 mg/dl or >5.55 mmol/l) or known diabetic patients, hypertriglyceridemia (>150 mg/dl or >1.65 mmol/l), high density lipoprotein (HDL)-cholesterol: (women < 50 mg/dl or <1.30 mmol/l), and abdominal obesity, as measured by a waist circumference of ≥88 cm for women. The research was carried out according to the principles of the declaration of Helsinki. The ethics committee of the Tehran University of Medical Science approved the study protocol. In the diabetes clinic, interviews were undertaken by physicians in order to obtain information about the patients’ history. Anthropometric measurements were conducted by well-trained examiners. Waist circumference (WC) was measured at the midpoint between the last rib and the iliac crest, directly on the skin. Weight was measured by a calibrated balance beam scale. BMI (kg/m^2^) was calculated according to the Quetelet formula. Blood pressure was measured with a standard calibrated mercury sphygmomanometer, applied to the right arm after 15 minutes of rest in the sitting position. In pre-menopausal subjects, 80.9% were being treated with one oral hypoglycemic agent and 19.1% received insulin therapy. In post-menopausal women, 75.4% received oral hypoglycemic agents and the other 24.6% received insulin as a glucose-lowering drug (Table 1). Enzymatic methods were used for the measurement of total cholesterol and triglycerides. Total cholesterol and high-density lipoprotein (HDL) cholesterol were measured by cholesterol oxidase phenol aminoantipyrine assays. IFCC methods were used to estimate human haemoglobin A1C. MetS was defined according to criteria from the NCEP ATP III [7], which defines MetS as the presence of at least three of the following: abdominal obesity as determined by an elevated WC of greater than 88 cm for women; a HDL cholesterol level lower than 50 mg/dl; triglyceride levels of 150 mg/dl or higher; systolic blood pressure of 130 mmHg or higher and diastolic blood pressure of 85 mmHg or higher or known hypertensive patients; diabetes mellitus. We also used IDF criteria with a recently defined local Iranian cut-off point for WC (≥85.5) according to Esteghamati et al. as an alternative measurement [8].

**Table 1:** **Baseline characteristics of study population**

	**Pre-menopause**	**Post-menopause**	**P-value**
	**(n = 221)**	**(n = 418)**	
Age (years)	43.33 ± 0.47	60.35 ± 0.38	< 0.001
Duration of diabetes (years)	6.21 ± 0.38	9.17 ± 0.35	< 0.001
Weight (Kg)	73.39 ± 0.95	71.84 ± 0.62	0.15
BMI (Kg/m^2^)	29.02 ± 0.35	28.75 ± 0.23	0.51
Uric acid (mg/dl)	4.34 ± 0.19	5.18 ± 0.22	< 0.05
Urea (mg/dl)	27.35 ± 0.95	31.06 ± 0.81	< 0.01
Creatinine (mg/dl)	0.85 ± 0.01	1.01 ± 0.03	< 0.005
HbA1C (%)	8.41 ± 0.18	8.40 ± 0.10	0.94
Post-prandial glucose (mg/dl)	265.79 ± 9.57	251.10 ± 6.80	0.21
Total Cholesterol (mg/dl)	185.76 ± 3.39	190.56 ± 2.63	0.27
LDL (mg/dl)	106.36 ± 3.83	103.43 ± 2.27	0.48
Triglycerides/HDL	4.23 ± 0.28	4.37 ± 0.21	0.71
Non-HDL cholesterol/HDL	3.19 ± 0.11	3.18 ± 0.08	0.91
AST (mg/dl)	22.04 ± 1.28	23.50 ± 1.43	0.52
ALT (mg/dl)	22.49 ± 1.36	24.69 ± 1.19	0.26
Albuminuria (mg/24 h)	8.17 ± 0.13	6.81 ± 0.07	0.21
Myocardial infarction (%)	3(1.3)	8(1.9)	0.49
Drugs (%)			
Glucose-lowering			0.14
Oral-hypoglycemic	80.9	75.4	
Insulin	19.1	24.6	
Lipid-lowering			0.28
Statin	83.5	91.3	
Fibrate	8.2	3.8	
Statin + Fibrate	4.1	1.9	
Anti-hypertensive			< 0.05
ACEI	32.6	22.6	
ARB	42.3	35.7	
Beta-blocker	15.3	21.4	
CCB	5.7	2.3	
Combined	3.8	17.8	

BMI, body mass index; HbA1C, hemoglobin A1C; LDL, low density lipoprotein cholesterol; HDL, high density lipoprotein cholesterol; AST, aspartate aminotransferase; ALT, alanine aminotransferase; eGFR, estimated glomerular filtration rate; ACEI, angiotensin converting enzyme inhibitor; ARB, aldosterone receptor blocker; CCB, calcium channel blocker.Note: topographic mean was represented for albuminuria.

### Statistical analysis

All data were analysed separately for pre-menopausal and post-menopausal groups. For continuous variables, mean ± SD was calculated. Data analysis was performed with the statistical package for the social sciences program (SPSS for windows, version 17; Chicago, IL). Student’s t-test and analysis of variance considering age and duration of diabetes as covariates were used to compare the variables between the two groups. Adjustments were made for all independent variables. Odds ratios with 95% CI were calculated by binary logistic regression analysis for factors affecting MetS. All analyses were two-tailed and a P-value of less than 0.05 was considered statistically significant.

## Results

MetS was defined for 88.3% of total subjects (87.5% and 87.7% in pre-menopausal and post-menopausal women with T2DM, respectively) (P = 0.34). Table 1 shows baseline characteristics of the study population. The participants were divided in two groups: 221 (34.6%) pre-menopausal women with T2DM and mean age of 43.33 ± 0.47 and 418 (65.4%) post-menopausal women with T2DM and mean age of 60.35 ± 0.38.

Comparisons of the MetS components in pre- and post-menopausal groups are presented in Table 2. There were significant differences in WC (P <0.01) and systolic HTN (P <0.001) between the two groups.

**Table 2:** **Metabolic syndrome components in pre- and post-menopause women with type 2 diabetes**

	**Pre-menopause**	**Post-menopause**	**P-value**
	**(n = 221)**	**(n = 418)**	
Waist (cm)	93.29 ± 0.9	96.17 ± 0.59	< 0.01
SBP (mmHg)	124.46 ± 1.42	131.42 ± 1.09	< 0.001
DBP (mmHg)	77.96 ± 0.88	78.41 ± 0.71	0.54
Triglycerides (mg/dl)	185.54 ± 9.07	190.42 ± 6.82	0.67
HDL (mg/dl)	47.08 ± 1.50	47.04 ± 0.81	0.98
FBS (mg/dl)	185.91 ± 5.24	183.65 ± 3.80	0.72

SBP, systolic blood pressure; DBP, diastolic blood pressure; HDL, high density lipoprotein; FBS, fasting blood sugar.

The association of MetS components with menopausal status in women with T2DM is shown in Table 3. In a multivariate logistic regression model with menopausal status as the outcome and MetS components as independent variables, there were significant differences in systolic HTN (odds ratio: 1.83, P <0.001) between the two groups (model 2). In model 3, menopausal status was defined as the outcome and metabolic syndrome components, duration of diabetes, BMI and haemoglobin A1C were defined as independent variables. SBP and WC differed significantly between the two groups.

**Table 3:** **Association of metabolic syndrome components with menopause status in women with type 2 diabetes**

		**Model 1**	**Model 2**	**Model3**
Waist	OR	1.26 (1.06 – 1.50)	1.13 (0.88 – 1.45)	1.56 (1.00 – 2.29)
	p-value	p <0.01	p = 0.32	p = 0.05
SBP	OR	1.39 (1.17 – 1.66)	1.83 (1.25 – 2.68)	1.63 (1.03 – 2.59)
	p-value	p <0.001	p <0.005	p <0.05
DBP	OR	1.05 (0.89 – 1.24)	0.70 (0.47 – 1.05)	0.88 (0.54 – 1.41)
	p-value	p = 0.54	p = 0.09	p = 0.60
Triglycerides	OR	1.04 (0.85 – 1.28)	1.02 (0.78 – 1.34)	0.97 (0.71 – 1.33)
	p-value	p = 0.67	p = 0.83	p = 0.88
HDL	OR	0.99 (0.80 – 1.23)	0.98 (0.76 – 1.26)	1.03 (0.75 – 1.42)
	p-value	p = 0.98	p = 0.91	0.82
FBS	OR	0.96 (0.80 – 1.15)	0.93 (0.73 – 1.19)	0.90 (0.64 – 1.25)
	p-value	p = 0.725	p = 0.61	p = 0.54

SBP, systolic blood pressure; DBP, diastolic blood pressure; HDL, high density lipoprotein; FBS, fasting blood sugar, OR, odds ratio.

Model 1: Univariate logistic regression model with menopause status as outcome variable.

Model 2: Multivariate logistic regression model with menopause status as outcome and metabolic syndrome components as independent variables.

Model 3: Multivariate logistic regression model with menopause status as outcome and metabolic syndrome components and duration of diabetes, body mass index and hemoglobin A1C as independent variables.

Note: Odds ratios were calculated for 1 standard deviation change in independent variable.

**Figure 1:**
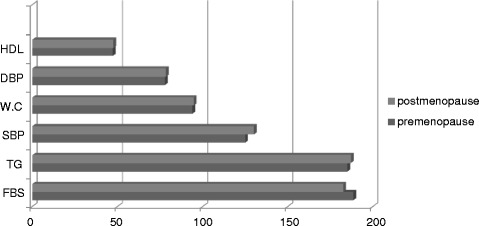
**The Met S Components according to ATP III Index in premenopausand post menopausal women with type II diabetes.** FBS: Fasting blood suger; WC: Waist circumference; HDL: High density lipoprotein; TG: Triglycerid; SBP: Systolic blood pressure; DBP: Diastolic blood pressure.

The components of MetS, according to the ATP III Index, in pre-menopausal and post-menopausal women with T2DM are shown in Figure 1.

## Discussion

In the present study, the differences between the components of MetS in post-menopausal women compared with pre-menopausal women were questioned, as T2DM was the underlying condition. There was no significant difference in MetS prevalence in pre-menopausal and post-menopausal women with T2DM. In the present study, WC and systolic blood pressure differed significantly between pre-menopausal and post-menopausal women with T2DM. Diastolic blood pressure, triglycerides, HDL-C and FBS did not differ between the two groups.

The frequency of MetS increases from the beginning of the menopausal transition to post menopause and the defined components of MetS are more frequent in non-diabetic, post-menopausal women compared to pre-menopausal women [1,9]. Ainy et al. [1] reported that the prevalence of MetS is 53% in pre-menopausal women and 69% in post-menopausal women [1]. This calls into question how the presence of T2DM reduces the differences observed between pre- and post-menopausal women.

Diabetes is an inflammatory state, characterised by increased plasma levels of reactive oxygen species, lipid per oxidation products, CRP, ferritin and other reactive compounds, which all increase with inflammatory process [10,11]. Furthermore, it has been shown that women with T2DM secrete higher levels of inflammatory factors such as Interleukin 6 (IL-6) and thus have a greater stress response than men [12].

In this study, HDL-C level did not differ in pre-menopausal compared with post-menopausal status in diabetic women. It has been proposed that in patients with T2DM, HDL is less effective at inhibiting oxidation and cholesterol efflux stimulation from macrophage [13,14]. In the presence of systemic inflammatory condition the atheroprotective effect of apolipoproteins and lipids may be lost; so that, for instance, high HDL-C plasma levels may be proatherogenic rather than protective [15]. Despite the loss of oestrogen during menopause, some studies have reported a gradual increase in HDL-C in women with T2DM [16]. In fact, menopause itself is not associated with a reduction in HDL-C levels, and there is a possibility that findings of low HDL-C levels in post-menopausal women in most previous reports may be due to weight gain, lack of physical activity and associated metabolic disease such as T2DM in older women [17].

In the present study, WC differed between pre-menopausal and post-menopausal groups. Abdominal obesity is one of the main clinical features of MetS in post-menopausal patients with T2DM [18]. Weight gain does not appear to be affected by the hormonal changes associated with the menopause [19]. Although increased visceral adiposity during menopausal transition is associated with increasing insulin resistance, elevated free fatty acid levels, as well as decreased adiponectin levels, have also been observed [2]. Studies have concluded that the average steady weight gain of about 0.5 kg annually observed in post-menopausal women, is caused by age rather than the menopause itself [19]. This suggests that increased WC in post-menopausal women may be caused by increasing age.

In the current study, post-menopausal women had higher systolic blood pressure compared to pre-menopausal women with T2DM (P <0.005) but there was no significant difference in diastolic blood pressure between the groups. Franklin et al. [20] showed that, in the general population, systolic blood pressure increases progressively with age, whereas diastolic blood pressure rises until 60 years of age, after which it starts to decline [20].

A twofold increase in CVD risk has been reported in post-menopausal women compared to pre-menopausal women [2]. However, in patients with diabetes, pre-menopausal women lose some protective factors observed in their healthy counterparts. A worsening of the plasma lipid profile may contribute to the loss of CVD protection seen in diabetic women [21,22]. Also, the pre-menopausal advantage in the clearance of dietary lipids in healthy subjects is not seen in pre-menopausal women with T2DM [21]. Another important finding is that in pre-menopausal women with T2DM, reduced coronary vasodilator function and impaired response of resistance vessels to increased sympathetic stimulation is observed, which is similar to that observed in non-diabetic post-menopausal women [23]. Besides the different mechanisms associated with diabetes, Aviles-Santa et al. [24] showed that risk of CVD in pre-menopausal women with T2DM who have multiple features of MetS is underestimated according to the Framingham scoring system.

Furthermore, diabetes can attenuate protective effects of oestrogen in premenopausal status [18]. Studies show that oestrogen deficiency appears to be associated with an increased risk of cardiovascular events [18,25]. In non-diabetic women, one of the beneficial effects of oestrogen in terms of the prevention of CVD and its risk factors is the increase in the basal release of nitric oxide (NO) from endothelial cells [15]. One of the possible explanations for the effects of the loss of oestrogen on vascular protection in diabetes is that oestrogen fails to affect basal NO release and alter vasodilation [15,26]. As a result, hormone replacement therapy (HRT) has demonstrated to cause both favourable changes in lipid profiles and the reduction of coronary heart disease in women with T2DM [27-30]. Greater benefits from HRT are expected if glucose metabolism has previously been normalised [22]. Moreover, the diabetic setting has been strongly associated with lower sex hormone binding globulin (SHBG) levels [29]. Lower SHBG level is considered to be detrimental to vasculature in women [29].

The main limitation of the present study was the cross-sectional study design used, which precludes the determination of the direction of causality. However, this is the first study to compare MetS in pre- and post-menopausal diabetic women.

In Conclusion, MetS must be evaluated in diabetic patients regardless of the time of menopausal transition [24]. In patients with diabetes, pre-menopausal women lose some protective factors observed in their healthy counterparts. In the present study, the pre-menopausal group suffered from the same incidence of MI as the post-menopausal group. Primary prevention through changes in lifestyle and diet and secondary prevention through early detection and good control of diabetes are necessary for the prevention of MetS and cardiovascular disease in Iranian women with T2DM, regardless of their menopausal status.

## References

[1] Ainy E, Mirmiran P, Zahedi Asl S (2007). Prevalence of metabolic syndrome during menopausal tromsition in Tehranian women: Tehran Lipid and Glucose Study (TLGS). Maturitas.

[2] Brown TM, Vaidya D, Rogers WJ (2008). Does prevalence of metabolic syndrome in women with coronary artery disease differ by the ATP III and IDF criteria?. J Womens Health.

[3] Ana C, Basso S, Maria R (2012). Prevalence of Metabolic syndrome and associated factors in women aged 35 to 65 years in Brazil. North Am Menopause Soc.

[4] Qader SS, Shakir YA, Nyberg P (2008). Sociodemographic risk factors of metabolic syndrome in middle-aged women, results from a population-based study of Swedish women, The women's Heath in the Lund Area (WHTLA) study. Climacteric.

[5] Eshtiaghi R, Esteghamati A, Nakhjavani M (2010). Menopause is an independent predictor of Metabolic syndrome in Iranian women. Maturitas.

[6] Wing RR, Mathews KA, Kuller LH (1991). Weight gain at the time of menopause. Arch Intern Med.

[7] National Cholesterol Education Program (NCEP) Expert Panel on Detection, Evaluation, and Treatment of High Blood Cholesterol in Adults (Adult Treatment Panel III) (2002). Third Report of the National Cholesterol Education program (NCEP) Expert panel on detection, evaluation and treatment of high blood cholesterol in adult final report. Circulation.

[8] Esteghamati AR, Ashraf H, Rashidi A (2008). Waist cicumference cut – off points for the diagnosis of metabolic syndrome in Iranian adults. Diabetes Res Clin Pract.

[9] Polotsky HN, Polotsky AJ (2010). Metabolic Implications of Menopause. Semin Reprod Med.

[10] Ross L, Polotsky A (2012). Metabolic correlates of menopause: an update. Curr Opin Obstet Gyneco.

[11] McGillicuddy FC, De La Liera Moya M (2009). Inflammation impairs reverse cholesterol transport in vivo. Circulation.

[12] Nakhjavani M, Morteza A (2011). Serum heat shock protein 70 and oxidized LDL in patients with type 2 diabetes :does sex matter?. Cell Stress Chaperones.

[13] Hoeg JM, Santamarina-Fojo S, Bérard AM, Cornhill JF, Herderick EE, Feldman SH, Haudenschild CC, Vaisman BL, Hoyt RF, Demosky SJ (1996). Overexpression of lecithin:cholesterol acyltransferase in transgenic rabbits prevents diet-induced atherosclerosis. Proc Natl Acad Sci U S A.

[14] Scanu AM, Edelstein C (2008). HDL :bridging past and present with a look at the future. FASEB J.

[15] Harris M, Hadden W, Knowler W (1987). Prevalence of diabetes and impaired glucose tolerance and plasma glucose levels in U.S. population aged 20–74 yr. Diabetes.

[16] Zethelius B, Eeg-Olofsson K, Nilsson PM, Gudbjörnsdottir S (2011). Blood lipids in 75 048 type 2 diabetic patients: A population-based survey from the Swedish National diabetes register. Eur J Cardiovasc Prev Rehabil.

[17] Ushiroyama T, Sakuma K, Keda AI (2005). The HDL2/HDL3 ratio in menopausal women. Int Gynaecol Obstet.

[18] Mesch VR, Bocro LE, Siseles N (2006). Metabolic syndrome Throughout the Menopausal transition: influence of age and menopausal status. Climacteric.

[19] Davis SR, Castelo-Branco C, Chedraui P (2012). Understanding weight gain at menopause. Climacteric.

[20] Franklin SS, Gustin W, Wong ND (1997). Hemodynamic patterns of age-related changes in blood pressure. Circulation.

[21] Masding MG, Stears AJ, Burdge GC (2003). Premenopausal Advantages in Postprandial Lipid Metabolism Are Lost in Women With Type 2 Diabetes. Diabetes Care.

[22] Schianca GPC, Colli E, Bigliocca M (2012). Sex difference in lipid profiles in relation to the progression of glucose abnormalities. J Diabetes.

[23] Di Carli MF, Afonso L, Campisi R (2002). Coronary vascular dysfunction in premenopausal women with diabetes mellitus. Am Heart J.

[24] Avilés-Santa L, Salinas K, Adams-Huet B (2006). Anthropometric feature and cardiovascular risk in young Latin Americans with type 2 diabetes mellitus. J Diabetes Complications.

[25] Johnstone MT, Creager SJ, Scales KM (1993). Impaired endothelium-dependent vasodilation in patients with insulin- dependent diabetes mellitus. Circulation.

[26] Fulton DJR, Hodgson WC, Sikorski BW (1991). Attenuated responses to endothelin- and KC1 and CaC12, but not noradrenaline, of aortae from rats with streptozotocin-induced diabetes mellitus. Br J Pharmacol.

[27] Newton KM, Lacroix AZ, Heckbert SR (2003). Estrogen Therapy and Risk of Cardiovasvular Events Among Women With Type 2 Diabetes. Diabetes Care.

[28] Lamon-Fava S, Herrington DM, Horvath KV (2010). Effect of hormone replacement therapy on plasma lipoprotein levels and coronary atherosclerosis progression in postmenopausal women according to type 2 diabetes mellitus status. Metabolism.

[29] Saltiki K, Cimponeriu A, Lili K (2008). Severity of coronary artery disease in postmenopausal diabetic women. Hormones.

[30] Bal S (1998). Use of hormone replacement therapy in women with diabetes. J Diab Nurs.

